# Author Correction: Heritable polygenic editing: the next frontier in genomic medicine?

**DOI:** 10.1038/s41586-025-08904-4

**Published:** 2025-03-21

**Authors:** Peter M. Visscher, Christopher Gyngell, Loic Yengo, Julian Savulescu

**Affiliations:** 1 Institute for Molecular Bioscience, University of Queensland, Brisbane, Australia; 2 Big Data Institute, Li Ka Shing Centre for Health Information and Discovery, Nuffield Department of Population Health, University of Oxford, Oxford, UK; 3 Murdoch Children’s Research Institute, Melbourne, Victoria Australia; 4 Department of Paediatrics, University of Melbourne, Melbourne, Australia; 5 Uehiro Oxford Institute, University of Oxford, Oxford, UK; 6 Centre for Biomedical Ethics, Yong Loo Lin School of Medicine, National University of Singapore, Singapore, Singapore

**Keywords:** Genetics research, Diseases, Genetic variation, Genetic predisposition to disease, Ethics

Correction to: *Nature* 10.1038/s41586-024-08300-4 Published online 8 January 2025

In the version of the article initially published, two simulation studies that modelled the effect of germline gene editing on human disease were omitted and have now been added as refs. 20 and 21: Oliynyk, R. T. Quantifying the potential for future gene therapy to lower lifetime risk of polygenic late-onset diseases. *Int. J. Mol. Sci.* 10.3390/ijms20133352 (2019) and Oliynyk, R. T. Future preventive gene therapy of polygenic diseases from a population genetics perspective. *Int. J. Mol. Sci.* 10.3390/ijms20205013 (2019). The authors regret this omission and have added a sentence to the main text and a paragraph in the revised Supplementary Note 1.

