# Supplementary material for: Heritable polygenic editing: the next frontier in genomic medicine?
Source: Nature. 2025 Jan 8;637(8046):637–45. doi: 10.1038/s41586-024-08300-4 (PMC11735401; doi:10.1038/s41586-024-08300-4)
Supplement: Supplementary file 1 — This file contains full descriptions for Supplementary Tables 1–3, Figs. 1–4, Box 1, Notes 1 and 2 and references. [file 41586_2024_8300_MOESM1_ESM.pdf]

nature portfolio

https://doi.org/10.1038/s41586-024-08300-4

Supplementary information

Heritable polygenic editing: the next frontier in genomic medicine?

In the format provided by the authors and unedited

Heritable polygenic editing as a potential frontier in genomic medicine

Peter M. Visscher, Christopher Gyngell, Loic Yengo, Julian Savulescu

TABLE OF CONTENTS

Supplementary Figures
Suppl. Fig. 1: Quantification of off-target effects of genome-editing on fitness.
Suppl. Fig. 2: Attenuation in outcome when 1, 2, or 5 out of the 10 variants have no effect on the phenotype.
Suppl. Fig. 3: Quantification of pleiotropic effects of genome-editing on fitness
Suppl. Fig. 4. Quantification of health inequality in a population including a fraction of genome-edited individuals

Supplementary Box
Suppl. Box 1: Single variant example

Supplementary Notes
Note 1. Benchmark calculations.
Note 2. Factors that attenuate actual outcomes or make them obsolete.

Supplementary Tables (additional data – spreadsheet)
Suppl. Tab 1. GWAS summary statistics for diseases analysed in this study. AD: Alzheimer's Disease; MDD: Major Depressive Disorder; SCZ: Schizophrenia; T2D: type 2 diabetes; CAD: Coronary Artery Disease.

Suppl. Tab 2. GWAS summary statistics for quantitative risk factors analysed in this study. FG: fasting glucose (estimated standard deviation (SD) = 0.55 mmol/L); LDL: LDL cholesterol levels (effect size is in SD of log LDL: estimated SD of log LDL = 1.11); TG: triglycerides levels (effect size is in SD of log TG: estimated SD of log TG = 1.10); SBP: Systolic Blood Pressure (estimated SD = 17.07 mmHg); SBP: Diastolic Blood Pressure (estimated SD = 10.70 mmHg).

Suppl. Tab 3. Summary statistics and results for the top-10 GWAS loci ranked on expected change after gene editing.

Supplementary References

SUPPLEMENTARY FIGURES

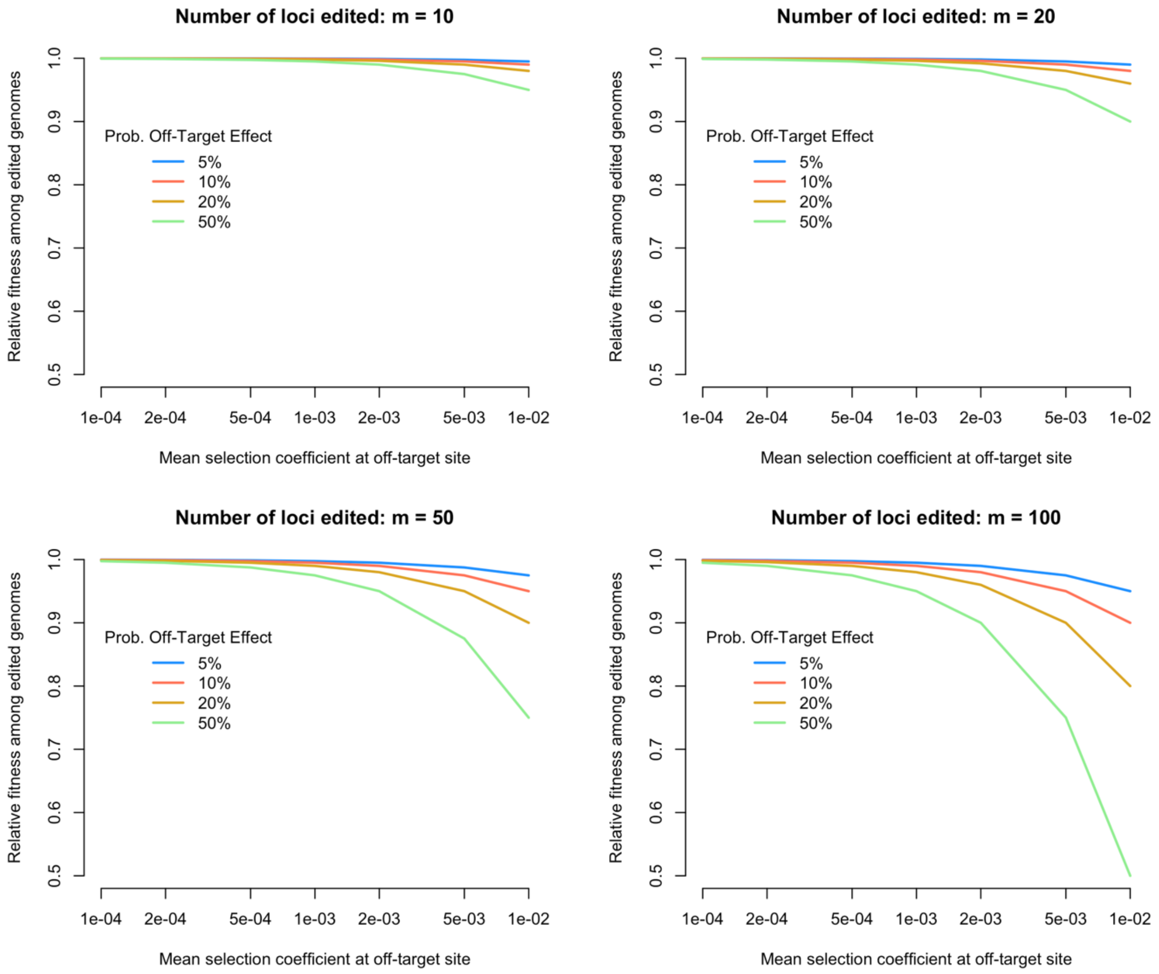

Suppl. Fig. 1. Quantification of off-target effects of genome-editing on fitness. We assumed a linear reduction in fitness caused by off-target of gene-editing: $w(m, s, p_O) = 1 - msp_O$, where p_O is the probability of off-target effects (varied here between 5% and 50%), m the total number of edited loci and s the average selection coefficient across all edited alleles. The source code to generate the Figure is provided in the **Code Availability** section in the main text.

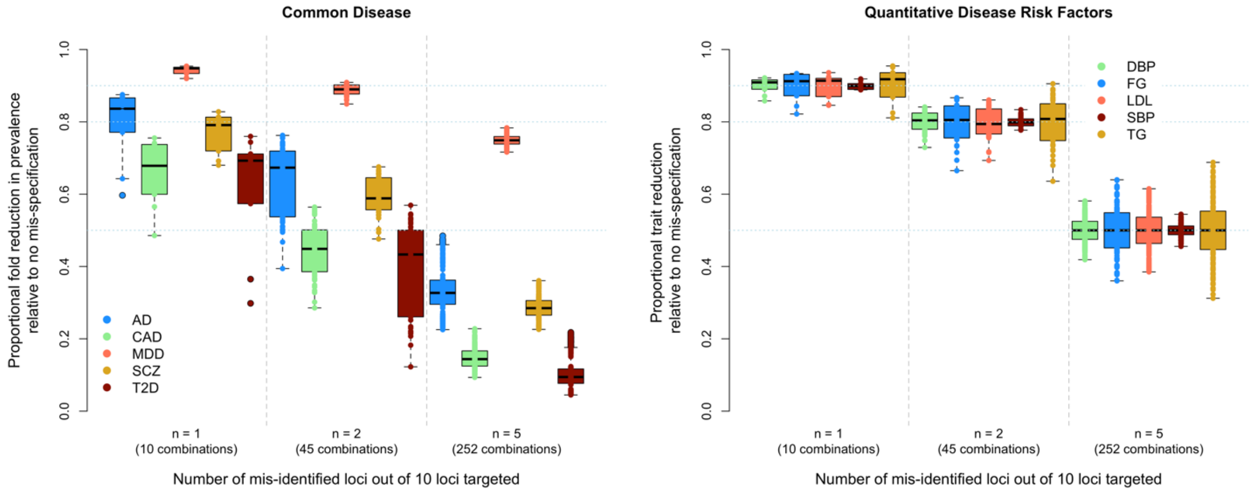

Suppl. Fig. 2. Attenuation in outcome when 1, 2, or 5 out of the 10 variants have no effect on the phenotype. We modelled this by assuming that 1, 2 or 5 of the 10 loci previously used in **Figure 1** were mis-specified and had no effect on the trait, enumerating all possible combinations (10 for 1 mis-specified locus, 45 for 2 mis-specified loci and 252 for 5 mis-specified loci). For quantitative traits we calculated the proportional reduction in actual outcome (in phenotypic SD units) compared to that outcome with no mis-specified loci. For disease, we calculated the proportional reduction in fold-change in prevalence relative to the case of no mis-specified loci. The source code to generate the Figure is provided in the **Code Availability** section in the main text.

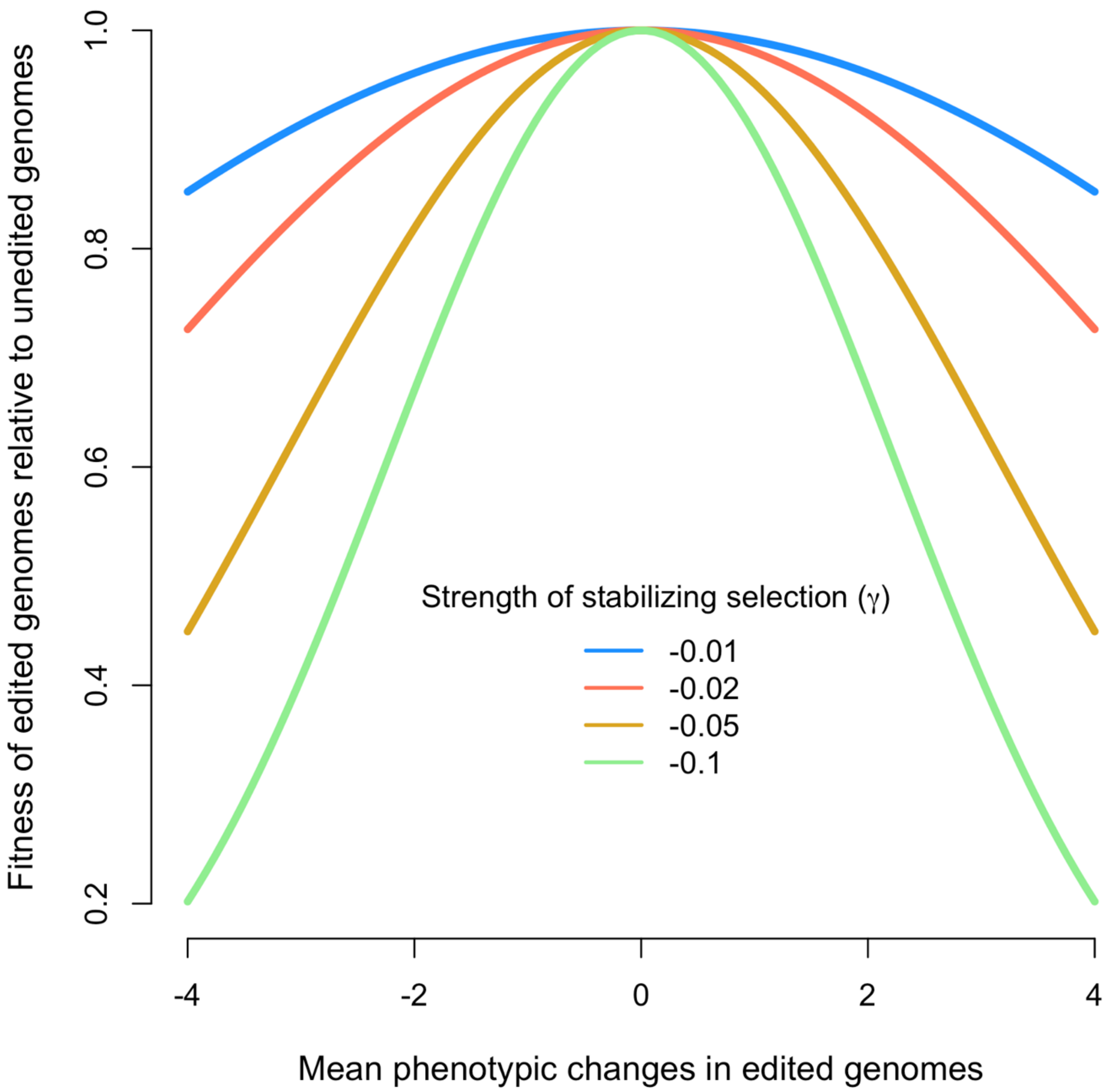

Suppl. Fig. 3. Quantification of pleiotropic effects of genome-editing on fitness. We modelled stabilising selection assuming a Gaussian fitness function and that the current population is at an optimum. Under this model, fitness is proportional to exp $[\gamma(y - y_{opt})^2]$, where γ is the quadratic regression coefficient from a regression of log(fitness) on phenotype and the squared phenotype. We varied γ from -0.01 to -0.1. The source code to generate the Figure is provided in the **Code Availability** section in the main text.

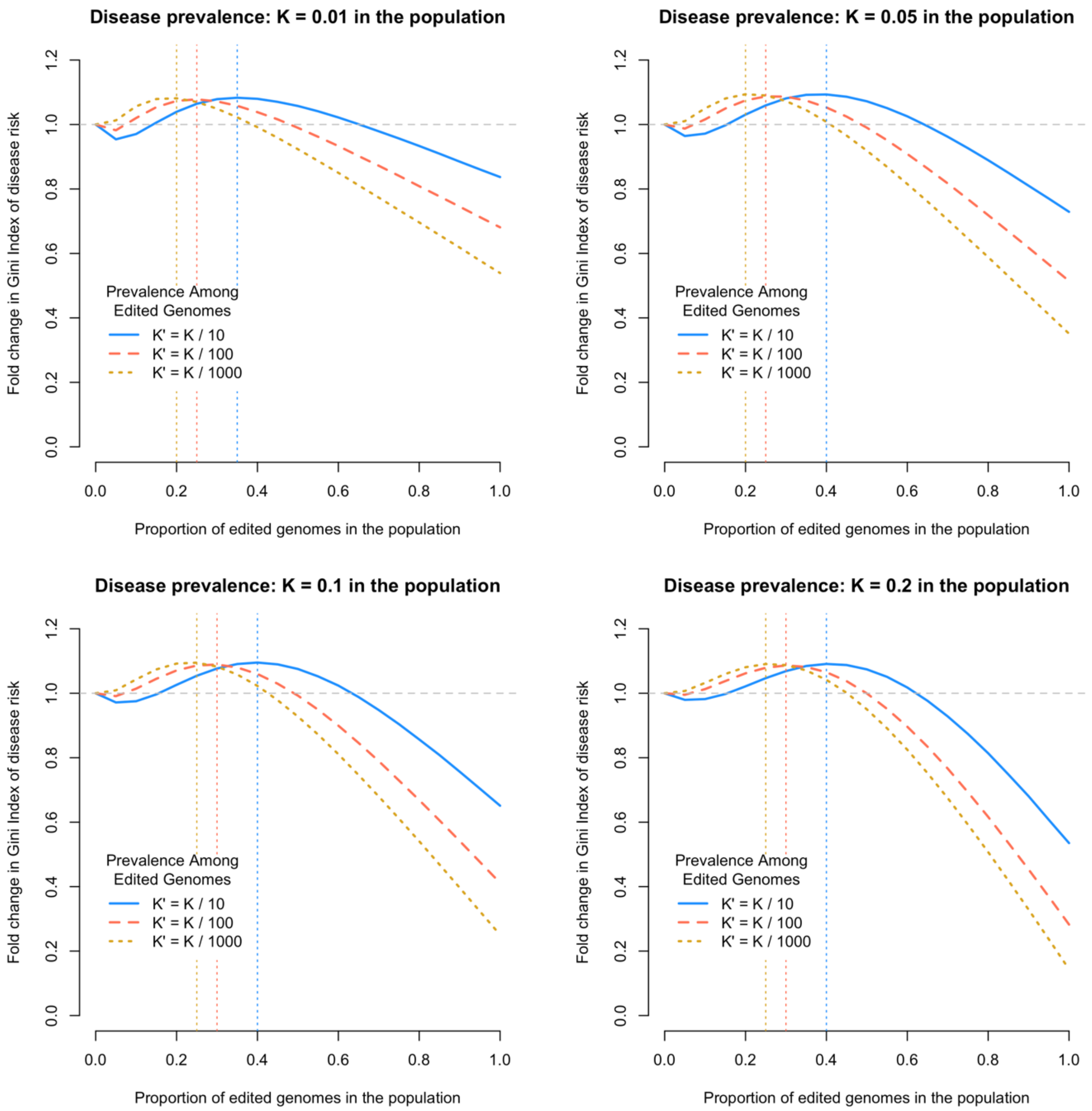

Suppl. Fig. 4. Quantification of health inequality in a population including a fraction of genome-edited individuals. We model the probability of disease in the population as a mixture distribution with two components: one component with a reduced risk, representing the fraction of edited genomes in the population, and another component representing unedited genomes. Each panel represents different disease prevalence: K =0.01, 0.05, 0.1 and 0.2. Within each panel, the x-axis represents the fraction of edited genomes in the population, varying from 0 to 1, and the y-axis represents the relative Gini index in the population as compared to a population with no edited genomes. Each line represents various editing strategies aiming at to achieve a 10 (blue plain line), 100 (coral dashed line) or 1000 (golden dotted line) -fold reduction in disease prevalence among edited genomes. The source code to generate the Figure is provided in the **Code Availability** section in the main text.

Supplementary Box 1: Single variant example

We consider the predicted effect of population-wide embryo screening or gene editing for a causal risk allele at a single locus. Assume a single locus multiplicative (on disease scale) model, with lifetime prevalence $K = f_0[1 + p(\lambda - 1)]^2$, with f_0 the probability of disease for an individual with no risk alleles at this locus, p the frequency of the risk allele and λ the relative risk (see **Methods**). After gene editing, the life-time prevalence in the population of edited genomes (K_g) is f_0, and the proportional reduction of disease prevalence after gene editing is $1 - K_g/K \ = 1 - 1/[1 + p(\lambda - 1)]^2$.

We contrast this with embryo screening to allow selection again the risk variant. We assume, optimistically, that enough embryos are screened so that a heterozygous parent always produces an embryo with their inherited non-risk allele. [The probability of not passing on a non-risk allele is $(1/2)^n$ for screening of n embryos, so 0.03 and 0.001 for n = 5 and n = 10, respectively. However, if both parents are heterozygous then the probability of not observing a homozygous non-risk genotype is $(3/4)^n$, which implies that 16 embryos are needed to reduce the chance of not observing the desired genotype to 1%]. We also assume Hardy-Weinberg equilibrium of genotype frequencies in the population, before selection. In couples with no segregating risk alleles there is no selection possible. This includes couples where both partners are homozygous (for either the risk or non-risk allele) and couples where one parent is homozygous for the risk allele and the other parent is homozygous for the non-risk allele. In total, a proportion of $[1 - 2p(1 - p)]^2$ couples are in these categories.

In the next generation after embryo selection, the frequency of homozygous risk, heterozygotes and homozygous non-risk genotypes in the population are p^4, $2p^2(1 - p^2)$ and $[1 - p^2]^2$, respectively, and the new frequency of the risk allele is p^2. Finally, the new lifetime prevalence in the population is calculated from the new genotype frequencies and their relative risks, and is derived as

$$K_s = K[1 + p^2(\lambda - 1)]^2/[1 + p(\lambda - 1)]^2 = f_0[1 + p^2(\lambda - 1)]^2$$

Therefore, the proportional reduction in lifetime prevalence because of gene editing relative to that of embryo screening is $[1 + p^2(\lambda - 1)]^{-2}$. This factor is smallest when the risk allele frequency is common and when its effect size is large. Two examples are given in the table below. For Alzheimer's Disease, a substantial reduction in lifetime prevalence is expected under either technology, because the effect size of *APOE* ε4 is large, and results are similar because the risk allele frequency is relatively low ($p = 0.14$). In contrast, gene editing is much more efficient than embryo screening for the very common schizophrenia risk variant ($p = 0.85$): the proportional reduction in prevalence is 27% for the former but only 4% for the latter. Embryo screening is not efficient because 52% (i.e., $p^4 = 0.85^4$) of couples are homozygous for the risk allele.

**Disease**	**Lifetime prevalence (K)**	**Locus (Allele)**	**Allele frequency (p)**	**Relative risk (λ)**	**K after gene editing**	**K after embryo selection**
Alzheimer's	5%	*APOE* (ε4)	0.14	3.2	2.90%	3.20%
Schizophrenia	1%	*rs115329265** (A)	0.85	1.2	0.73%	0.96%

**rs115329265* is the SNP most significantly associated with schizophrenia at the major histocompatibility complex. There is no evidence currently that this SNP has a causal role for schizophrenia.

Supplementary Note 1: Benchmark calculations

Introduction

In this section we provide benchmark calculations on what might be achievable when editing multiple causal variants for a specific complex trait. For our calculations we assume, speculatively, that future reproductive and gene editing technologies allow precise genome editing at multiple loci in germ cells, and that resulting embryos, with genomes edited to be homozygous for desired alleles at those loci, are viable. We also assume that the identified loci are functional (causal) in their effect on the complex trait. While these assumptions currently cannot be fulfilled, we believe it is reasonable to assume they will be in the future. We derive predictions for several common diseases and quantitative traits, as a function of the number of edited loci.

Methods

We first quantify the effect of gene editing on a quantitative trait and subsequently on the prevalence of common disease.

Gene editing and quantitative traits

m: number of genome-wide significant (GWS) loci, with functional variants identified
p_i: allele frequency of the trait increasing allele at SNP i ($i = 1, \dots, m$)
x_i: number of trait increasing alleles (0, 1, 2) at SNP i
β_i: effect size of trait increasing allele (at SNP i) in phenotypic (or liability) standard deviation units

Under the assumption of Hardy-Weinberg equilibrium (HWE), $\mathrm{E}[x_i] = 2p_i$ and $\mathrm{var}[x_i] = 2p_i(1 - p_i)$.

The mean in the population can be represented as,

$$\mathrm{E}[Y] = \mu + \sum_{i=1}^{m} 2p_i,$$

with μ a constant. If germline gene editing were to be applied to all m loci, by making all loci homozygous for either the increasing alleles (all x_i = 2, so p_i = 1) or the decreasing allele (all x_i = 0, so p_i = 0), the expected phenotype of the gene-edited genome become(s),

$$\mathrm{E}[Y_{\mathrm{high}}] = \mu + \sum_{i=1}^{m} 2\beta_i$$

and $\mathrm{E}[Y_{\mathrm{low}}] = \mu$.

Hence the difference between the current mean and the predicted mean after gene editing is

$$\mathrm{E}[\mathrm{increase}] = \sum_{i=1}^{m} 2(1 - p_i)\beta_i$$

and

$$\mathrm{E}[\mathrm{decrease}] = -\sum_{i=1}^{m} 2p_i\beta_i,$$

respectively.

These calculations are from assuming that we replace an 'average' genome with a genome that contains all desirable alleles at the known loci, everything else held constant. Any specific genome will deviate from the average and therefore the predicted change in phenotype will depend on the sample of genomes that are edited. However, for many GWS loci this variance is small relative to the mean. If, for example, increasing alleles at m independent GWS loci all have a frequency of p, then the mean and variance of the number of increasing alleles per genome is $2mp$ and $2mp(1 - p)$, respectively. Hence the coefficient of variation is proportional to $1/\sqrt{m}$.

More generally, if the phenotypic variance explained by the m loci in the current population is $q^2 = \sum_{i=1}^{m} 2p_i(1-p_i)\beta_i^2$, then the standard deviation in phenotypic change among genomes is q. If we express this as a proportion of the absolute value of the mean predicted change, then the relative standard deviation is $q/[\sum_{i=1}^{m} 2(1-p_i)\beta_i]$ and $q/[\sum_{i=1}^{m} 2p_i\beta_i]$ for an increase and decrease in phenotypic change, respectively. For a single locus, these ratios are $\sqrt{p/[2(1-p)]}$ and $\sqrt{(1-p)/(2p)}$, respectively.

The standardised variance of the future phenotype of the edited genome(s) is approximately $(1-q^2)$. This is similar to the phenotype variance before editing, assuming that the GWS loci do not explain a large proportion of the variance among unedited genomes.

Gene editing and disease

We consider two multi-locus models, the liability threshold model and the multiplicative risk model[1,2].

Multiplicative model

Following refs.[1,2], this model is multiplicative on the risk scale both within and between loci, and therefore additive on the scale of log-risk. For a single locus, the probability of disease (D) conditional on the number of risk alleles X can be expressed as

$$\mathrm{P}(\mathrm{D}|X=x) = f_0\lambda^x$$

where the distribution X is 0, 1, 2 follows HWE probabilities. Then

$$K = \mathrm{E}[\mathrm{P}(D|X)] = f_0[1+p(\lambda-1)]^2$$

With gene editing, x = 0 (risk alleles) and $\mathrm{E}[\mathrm{P}(D|X=0)] = K_g = f_0$.

Therefore, the proportional reduction of disease after gene editing is

$$1 - K_g/K = 1 - 1/[1+p(\lambda-1)]^2 \approx 1 - \exp[-2p(\lambda-1)] \approx 2p(\lambda-1),$$

the latter approximation assuming that $p(\lambda-1)$ is small. Even when editing a single locus, the reduction can be substantial. Editing a common risk variant with small effect, for example $p = 0.3$ and $\lambda = 1.05$, is predicted to reduce prevalence by 3% (see also **Supplementary Box 1**).

If there are m (independent) loci with the same risk allele frequency and effect size, then

$$K = f_0[1+p(\lambda-1)]^{2m}$$

and

$$1 - K_g/K = 1 - 1/[1+p(\lambda-1)]^{2m} \approx 1 - \exp[-2mp(\lambda-1)] \approx 2mp(\lambda-1).$$

The last approximation breaks down when $p(\lambda-1) > 1/(2m)$.

This equation is valid even if the m loci are only a subset of all risk loci that are segregating in the population (e.g. the m loci are from GWAS), because the contribution from all other loci is contained in the parameter f_0. Allowing the allele frequencies and effect sizes to vary across loci leads to

$$K = f_0\mathrm{E}\left[\prod_{i=1}^{m}\lambda_i^{x}\right] \approx f_0\left[\prod_{i=1}^{m}[1+p_i(\lambda_i-1)]^2\right]$$

and

$$1 - K_g/K \ = 1 - 1/\mathrm{E}\left[\prod_{i=1}^{m} \lambda_i^x\right] \approx 1 - \prod_{i=1}^{m}[1 + p_i(\lambda_i - 1)]^{-2}.$$

If all $p_i(\lambda_i - 1)$ terms are very small, then

$$1 - \frac{K_g}{K} \approx \sum_{i=1}^{m} 2p_i(\lambda_i - 1),$$

with the same caveat about this approximation as before.

The assumptions of this model are expected to hold, because we propose that the gene editing is only attempting to access a small proportion of loci contributing to genetic risk. The model breaks down if there are variants explaining a high proportion of the genetic variance.[3] Under these circumstances the liability threshold model is better.

Liability threshold model

Liability (ℓ) for (multi-locus) genotype g is defined as $\ell = \mathrm{g} + \mathrm{e}$, with $\mathrm{E}[\ell] = \mathrm{E}[\mathrm{g}] = \mathrm{E}[\mathrm{e}] = 0$ and $\mathrm{var}[\ell] = 1$. The probability of disease (D) given genotype (G) can be expressed as

$$\mathrm{P}(\mathrm{D}|G = \mathrm{g}) = 1 - \Phi[t - \mathrm{g}] \text{ and such that } \mathrm{E}[\mathrm{P}(\mathrm{D}|G)] = 1 - \Phi(t) = K,$$

where Φ denotes the cumulative normal density function and t the threshold corresponding to lifetime prevalence K. Let z be the height of the density function pertaining to t. For a single locus,

$$\mathrm{E}[\mathrm{P}(\mathrm{D}|G, X)] = 1 - \mathrm{E}[\Phi(t - (X - 2p)\beta)] = K$$

with X = 0, 1, 2, $\mathrm{E}[X] = 2p$ and β the effect (in standard deviation units on the liability scale) of the risk increasing variant. After gene editing, X = 0 for the target variants and

$$K_g = \ \mathrm{E}[\mathrm{P}(\mathrm{D}|G, X = 0)] = 1 - \Phi(t + 2p\beta) \approx K - 2pz\beta,$$

which leads to

$$1 - K_g/K \ = 1 - [1 - \Phi(t + 2p\beta)]/K \approx 2pz\beta/K.$$

For multiple loci with equal effect size and allele frequencies,

$$K_g = \ \mathrm{E}[\mathrm{P}(\mathrm{D}|G, X_i = 0)] = 1 - \Phi(t + 2mp\beta) \approx K - 2mpz\beta,$$

with the constraint that $\beta \ll K/(2mpz)$. For the general case of unequal effect sizes and allele frequencies of risk variants,

$$\boxed{K_g = \ \mathrm{E}[\mathrm{P}(\mathrm{D}|G, X_i = 0)] = 1 - \sum_{i=1}^{m} \Phi(t + 2p_i\beta_i).}$$

This is the equation that was used to generate **Figure 1** in the main text.

Relationship between the models

For a single locus, the proportional reductions in disease are $2p(\lambda - 1)$ for the log(risk) model and $2pz\beta/K$ for the liability threshold model. Hence $\lambda = 1 + z\beta/K$. This can also be seen by assuming that the liability threshold model is the true model and then calculating the relative risks.

For each of the 3 genotypes,
$E[P(D|G, X = 0)] = 1 - \Phi(t + 2p\beta - 0\beta) \approx K - 2pz\beta$
$E[P(D|G, X = 1)] = 1 - \Phi(t + 2p\beta - 1\beta) \approx K - 2pz\beta + z\beta$
$E[P(D|G, X = 2)] = 1 - \Phi(t + 2p\beta - 2\beta) \approx K - 2pz\beta + 2z\beta$

Hence the relative risks are:

$$\frac{E[P(D|G, X = 1)]}{E[P(D|G, X = 0)]} = 1 + \frac{z\beta}{[K - 2pz\beta]} \approx 1 + z\beta/K$$

and also, approximately, $E[P(D|G, X = 2)]/E[P(D|G, X = 1)] \approx 1 + z\beta/K$.

Ref.[4] (Supplementary information) assumed and Ref.[5] showed that the genetic variance on the liability scale is approximately $2p(1-p)(\mathrm{GRR}-1)^2/i^2$, with GRR being the genotype relative risk, assumed to be multiplicative, and $i = z/K$. Therefore, the implicit effect size on the liability scale was $\beta = (\mathrm{GRR} - 1)/i\ = (\lambda - 1)K/z$, hence $\lambda = 1 + z\beta/K$, the same as above.

Empirical data are consistent with multiplicative models of genetic and environmental factors on the observed prevalence scale and (therefore) additivity on logistic or liability scales (e.g., ref.[6]).

Numerical examples

We provide numerical examples for editing 1 or 2 loci for LDL and T2D. Details about those loci are found in **Supplementary Table 3**. For these examples we round the values of the allele frequencies and effects sizes.

For LDL, the increasing alleles for the top 2 loci (ranked on their predicted effect of gene editing) have frequencies of 0.9998 and 0.9999 with effect sizes of 0.87 and 0.86 phenotypic standard deviations (**Supplementary Table 3**). The allele frequencies are for genetically inferred individuals of European ancestry. Assuming that the desired direction is to decrease the expected phenotype through gene editing, the expected phenotypic values before and after gene editing the first locus are μ + 2(0.9998)0.87 and μ + 2(0)0.87, respectively, giving a difference of -1.74 SD. Likewise, for the second locus, the difference is -1.72 SD and the cumulative difference is -3.46 SD. These predicted changes are very large because the desired trait-decreasing alleles are very rare, with frequencies of 0.0002 and 0.0001, respectively, yet have large effect sizes.

For T2D we assume a liability threshold model and a prevalence among unedited genomes of 10%, giving a threshold of 1.2816 on the scale of liability. The risk allele frequencies are 0.994 and 0.983 with effect sizes of 0.200 and 0.167, respectively (**Supplementary Table 3**). The reduction in liability, using the same equation as for LDL, is -0.40 and -0.33 for the first two loci, and cumulatively -0.73. These are converted to the prevalence scale as 1-Φ(1.28 – 0.20), 1-Φ(1.28 – 0.33) and 1-Φ(1.28 – 0.73) when editing the first locus, the second loci or both, resulting in predicted prevalences of 4.6%, 5.4% and 2.2%, and fold-reductions of 10/4.6 = 2.2, 1.9 and 4.5, respectively.

Violation of the simplified assumptions

The theoretical results shown are "best case scenarios" under a large number of assumptions, including (i) error-free gene editing technology with 100% efficacy, (ii) perfectly identified causal trait variants, (iii) additive genetic effects at causal variants, (iv) absence of G × E and G × G interactions, (v) no changes in future environments, (vi) no modelled deleterious pleiotropic effects. They give a benchmark for discussion but are known be overly simplified and approximate. In this section we discuss and quantify the effects of relaxation of these assumptions. Such quantification involves additional assumptions and parameters and are therefore also speculative.

Violating the assumptions can lead to the trait-specific mean among editing genomes ("gain") being less than that predicted and/or lead to undesirable effects on other traits. We discuss and model those each in turn in **Supplementary Note 2**.

Prior modelling

In a series of simulation studies, Oliynyk[7,8] explored the effect of preventative (prophylactic) gene therapy on human disease. Preventative gene therapy in the Oliynyk papers is the same as what we termed "heritable polygenic editing". Oliynyk assumed genetic architectures (the combination of risk allele frequences and effect sizes) and calibrated the number of simulated loci to achieve a given heritability. In the first study[7], age of onset profiles of several late-onset diseases and cancers were modelled, and the second study modelled a hypothetical disease with a heritability of 0.5 and a multiplicative genetic architecture of risk[8]. The effects of preventative gene therapy on disease prevalence and age of onset profiles were quantified by changing polygenic risk scores of individuals in the entire population by an assumed fixed proportion, for example by a 4-fold reduction in hazards ratio[7] or a 10-fold reduction in multiplicative risk[8].

In these papers, the effect of gene editing is given as input, which fundamentally differs from our approach. For a one-generation perspective, modelling the effect as a given input is no different to any environmental or therapeutic intervention that achieves the same imposed change in disease risk in the population[7]. The assumed genetic architectures in Oliynyk do not include rare protective effects, which are predicted to have the largest effect of reduced risk among edited genomes. In contrast, the mathematical modelling in this paper starts with empirical genetic architectures derived from GWAS for a number of complex traits and diseases, which include rare protective variants, and quantifies the effect of editing alleles at one or more loci on disease prevalence and trait means, with loci ordered on their predicted effect. Hence, the effect of gene editing is a result (output) of the modelling.

Supplementary Note 2: Factors that attenuate actual outcomes or make them obsolete

In this note we discuss a number of factors that would lead to the actual outcomes of HPE less than predicted or make HPE obsolete.

1. Environmental changes. Future environmental effects can cause phenotypes to differ greatly from that predicted from gene editing. New therapies could prevent or cure disease or reduce their prevalence. Therapeutic (somatic) gene editing could become so successful that there would be no justification for germline gene editing. It is hard to generalise or quantify such innovations, but we note that there are successful examples such as diet and PKU. Results from epidemiology suggests, broadly, that treatment effects on common chronic diseases are not large (odds ratios < 2).

2. Reduced efficacy of multi-locus gene editing and causal variant mis-identification. If only a proportion of targeted causal variants are successfully edited, either because the technology is imperfect or the actual causal variants are mis-identified, then the actual phenotypic changes among edited genomes will be less than predicted (or deleterious, discussed below). We modelled this by assuming that 1, 2 or 5 of the 10 loci previously used in **Figure 1** were mis-specified and had no effect on the trait, enumerating all possible combinations (10 for 1 mis-specified locus, 45 for 2 mis-specified loci and 252 for 5 mis-specified loci). For quantitative traits we calculated the proportional reduction in actual outcome (in phenotypic SD units) compared to that outcome with no mis-specified loci. For disease, we calculated the proportional reduction in fold-change in prevalence relative to the case of no mis-specified loci (**Supplementary Figure 2**).

3. GxE and GxG. Interactions between genomes and (future) environments (G × E) or interactions between specific variants and the genomic background (G × G) will both cause a reduction in phenotypic effect compared to the prediction. We modelled a reduction in actual phenotypic changes compared to that predicted by assuming that the genetic correlation r_g between the future phenotype and edited genome is less than 1 (**Figure 2** in main text).

Factors that give undesirable effects

4. Off-target deleterious effects. Off-target effects may not be neutral but have a deleterious effect on individuals with edited genomes. We modelled this by assuming a probability of an edited site creating an off-target effect with an effect on fitness. We assume that this reduction in fitness is irrespective of which loci and therefore which trait being targeted and that fitness is additive between loci. In particular, we assumed a linear reduction in fitness caused by off-target of gene-editing: $w(m, s, p_O) = 1 - msp_O$, where p_O is the probability of off-target effects (varied here between 5% and 50%), m the total number of edited loci and s the average selection coefficient across all edited alleles (**Supplementary Figure 1**).

5. Pleiotropy. Pleiotropy is ubiquitous in nature and therefore it is likely that change in one trait due to gene editing will have effects on other traits. Rather than model this for any specific correlated trait, we tried to be more general and model the negative effect of changing the mean of any trait on fitness. In particular, we modelled stabilising selection assuming a Gaussian fitness function and that the current population is at an optimum. Under this model, fitness is proportional to exp $[\gamma(y - y_{opt})^2]$, where γ is the quadratic regression coefficient from a multiple regression of log(fitness) on phenotype and the squared phenotype. Under stabilising selection and a Gaussian fitness function, $\gamma = -1/2V_S$, with V_S a variance term describing the strength of stabilising selection[9,10]. From contemporary human data, significant values of γ for a number of quantitative traits that were consistent with stabilising selection were in the range -0.01 to -0.05, implying weak to moderate selection[11] (**Supplementary Figure 3**).

Remark on human evolution

If HPE were to be applied in a country or population, then it could lead to a directional evolutionary change if, on average, genomes are edited for the same (combination of) complex traits. How much change will depend on the proportion of children born with edited genomes, the number of loci in their genomes that were edited and the effect sizes on traits and fitness at those loci. Mean phenotypic change for more than a single generation will additionally depend on the lifetime reproductive success of individuals with and without edited genomes. Making predictions on evolutionary change is even more speculative than predicting phenotypic changes among a sample of genomes edited at the same loci. Qualitatively, we can conclude that evolutionary change could be sufficiently large for it to be observable in only a few generations, because in the absence of germline manipulation to change allele frequencies in a directional manner, natural selection can change frequencies at trait-associated loci directionally in relatively short time periods. For example, it was reported recently that polygenic adaptation at multiple complex traits in the last 2000–3000 years has shaped genotypic and phenotypic variation in modern humans [12], and that loci associated with educational attainment have been under selection in contemporary populations in the UK and Iceland in the last 3 to 4 generations, leading to a reduction in the average predicted genetic value for this trait [13,14].

SUPPLEMENTARY REFERENCES

1 Risch, N. Linkage strategies for genetically complex traits. I. Multilocus models. *Am J Hum Genet* **46**, 222-228 (1990).

2 Wray, N. R., Goddard, M. E. & Visscher, P. M. Prediction of individual genetic risk to disease from genome-wide association studies. *Genome Res* **17**, 1520-1528 (2007).

3 Wray, N. R. & Goddard, M. E. Multi-locus models of genetic risk of disease. *Genome Med* **2**, 10 (2010). https://doi.org/10.1186/gm131

4 Purcell, S. M. *et al.* Common polygenic variation contributes to risk of schizophrenia and bipolar disorder. *Nature* **460**, 748-752 (2009). https://doi.org/Doi 10.1038/Nature08185

5 Yang, J., Wray, N. R. & Visscher, P. M. Comparing apples and oranges: equating the power of case-control and quantitative trait association studies. *Genet Epidemiol* **34**, 254-257 (2010). https://doi.org/10.1002/gepi.20456

6 Pang, S. *et al.* Genetic and modifiable risk factors combine multiplicatively in common disease. *Clin Res Cardiol* **112**, 247-257 (2023). https://doi.org/10.1007/s00392-022-02081-4

7 Oliynyk, R. T. Quantifying the Potential for Future Gene Therapy to Lower Lifetime Risk of Polygenic Late-Onset Diseases. *Int J Mol Sci* **20** (2019). https://doi.org/10.3390/ijms20133352

8 Oliynyk, R. T. Future Preventive Gene Therapy of Polygenic Diseases from a Population Genetics Perspective. *Int J Mol Sci* **20** (2019). https://doi.org/10.3390/ijms20205013

9 Walsh, B. & Lynch, M. *Evolution and selection of quantitative traits*. (Oxford University Press, 2018).

10 Johnson, T. & Barton, N. Theoretical models of selection and mutation on quantitative traits. *Philos Trans R Soc Lond B Biol Sci* **360**, 1411-1425 (2005). https://doi.org/10.1098/rstb.2005.1667

11 Sanjak, J. S., Sidorenko, J., Robinson, M. R., Thornton, K. R. & Visscher, P. M. Evidence of directional and stabilizing selection in contemporary humans. *Proc Natl Acad Sci U S A* **115**, 151-156 (2018). https://doi.org/10.1073/pnas.1707227114

12 Field, Y. *et al.* Detection of human adaptation during the past 2000 years. *Science* **354**, 760-764 (2016). https://doi.org/10.1126/science.aag0776

13 Beauchamp, J. P. Genetic evidence for natural selection in humans in the contemporary United States. *Proc Natl Acad Sci U S A* **113**, 7774-7779 (2016). https://doi.org/10.1073/pnas.1600398113

14 Kong, A. *et al.* Selection against variants in the genome associated with educational attainment. *Proc Natl Acad Sci U S A* **114**, E727-E732 (2017). https://doi.org/10.1073/pnas.1612113114
