# Supplementary material for: Heritable polygenic editing: the next frontier in genomic medicine?
Source: Nature. 2025 Jan 8;637(8046):637–45. doi: 10.1038/s41586-024-08300-4 (PMC11735401; doi:10.1038/s41586-024-08300-4)
Supplement: Supplementary file 2 — Reporting Summary [file 41586_2024_8300_MOESM2_ESM.pdf]

nature portfolio

Corresponding author(s): Peter M Visscher and Julian Savulescu

Last updated by author(s): Oct 4, 2024

Reporting Summary

Nature Portfolio wishes to improve the reproducibility of the work that we publish. This form provides structure for consistency and transparency in reporting. For further information on Nature Portfolio policies, see our Editorial Policies and the Editorial Policy Checklist.

Statistics

For all statistical analyses, confirm that the following items are present in the figure legend, table legend, main text, or Methods section.

n/a	Confirmed	
☐	☒	The exact sample size (*n*) for each experimental group/condition, given as a discrete number and unit of measurement
☒	☐	A statement on whether measurements were taken from distinct samples or whether the same sample was measured repeatedly
☒	☐	The statistical test(s) used AND whether they are one- or two-sided<br>*Only common tests should be described solely by name; describe more complex techniques in the Methods section.*
☒	☐	A description of all covariates tested
☐	☒	A description of any assumptions or corrections, such as tests of normality and adjustment for multiple comparisons
☐	☒	A full description of the statistical parameters including central tendency (e.g. means) or other basic estimates (e.g. regression coefficient) AND variation (e.g. standard deviation) or associated estimates of uncertainty (e.g. confidence intervals)
☒	☐	For null hypothesis testing, the test statistic (e.g. *F*, *t*, *r*) with confidence intervals, effect sizes, degrees of freedom and *P* value noted<br>*Give P values as exact values whenever suitable.*
☒	☐	For Bayesian analysis, information on the choice of priors and Markov chain Monte Carlo settings
☒	☐	For hierarchical and complex designs, identification of the appropriate level for tests and full reporting of outcomes
☒	☐	Estimates of effect sizes (e.g. Cohen's *d*, Pearson's *r*), indicating how they were calculated

Our web collection on statistics for biologists contains articles on many of the points above.

Software and code

Policy information about availability of computer code

Data collection	GWAS summary statistics were downloaded from the papers cited in the manuscript.
Data analysis	Data and R scripts underlying all Figures are available on GitHub (https://github.com/loic-yengo/Code_for_GeneEditing_Paper_Visscher_et_al_2024). Allele frequencies of SNPs significantly associated with Major Depression Disorder, Alzheimer's Disease and Schizophrenia used in our study were calculated using PLINK v1.9 from a set of 348,501 unrelated European ancestry participants of the UK Biobank (as previously described - Yengo et al. HMG (2018)). Analyses were performed using R version 4.2.0.

For manuscripts utilizing custom algorithms or software that are central to the research but not yet described in published literature, software must be made available to editors and reviewers. We strongly encourage code deposition in a community repository (e.g. GitHub). See the Nature Portfolio guidelines for submitting code & software for further information.

Data

Policy information about availability of data

All manuscripts must include a data availability statement. This statement should provide the following information, where applicable:

- Accession codes, unique identifiers, or web links for publicly available datasets
- A description of any restrictions on data availability
- For clinical datasets or third party data, please ensure that the statement adheres to our policy

All GWAS summary statistics used in the paper are available in the public domain. Data (i.e., specific sets of SNP, effect sizes and frequencies) and R scripts

underlying all Figures are publicly available on GitHub (https://github.com/loic-yengo/Code_for_GeneEditing_Paper_Visscher_et_al_2024). UK Biobank data were accessed under application 12505.

Human research participants

Policy information about studies involving human research participants and Sex and Gender in Research.

Reporting on sex and gender	N/A
Population characteristics	Allele frequencies of SNPs significantly associated with Major Depression Disorder, Alzheimer's Disease and Schizophrenia used in our study were calculated using PLINK v.19 from a set of 348,501 unrelated European ancestry participants of the UK Biobank (as previously described - Yengo et al. HMG (2018)).
Recruitment	UK Biobank investigators sent postal invitations to 9,238,453 individuals registered with the UK's National Health Service who were aged 40–69 years and lived within approximately 25 miles (40 km) of one of 22 assessment centers located throughout England, Wales, and Scotland. Overall, 503,317 participants consented to join the study cohort and visited an assessment center between 2006 and 2010, resulting in a participation rate of 5.45%. (Fry et al. Am J Epidemiol. 2017 Nov 1;186(9):1026-1034).
Ethics oversight	The National Information Governance Board for Health and Social Care and the North West Multicentre Research Ethics Committee provided approval for UK Biobank to obtain the contact details of people within the eligible age range from local National Health Service Primary Care Trusts.

Note that full information on the approval of the study protocol must also be provided in the manuscript.

Field-specific reporting

Please select the one below that is the best fit for your research. If you are not sure, read the appropriate sections before making your selection.

☒ Life sciences ☐ Behavioural & social sciences ☐ Ecological, evolutionary & environmental sciences

For a reference copy of the document with all sections, see nature.com/documents/nr-reporting-summary-flat.pdf

Life sciences study design

All studies must disclose on these points even when the disclosure is negative.

Sample size	Allele frequencies of SNPs significantly associated with Major Depression Disorder, Alzheimer's Disease and Schizophrenia used in our study were calculated using PLINK v.19 from a set of 348,501 unrelated European ancestry participants of the UK Biobank (as previously described - Yengo et al. HMG (2018)).
Data exclusions	We used two exclusion criteria for calculating allele frequencies: (1) Non-European ancestry individuals and (2) related individuals. Ancestry and relatedness was inferred using called genotypes at imputed SNPs as previously described in Yengo et al. (2018).
Replication	N/A
Randomization	N/A
Blinding	N/A

Reporting for specific materials, systems and methods

We require information from authors about some types of materials, experimental systems and methods used in many studies. Here, indicate whether each material, system or method listed is relevant to your study. If you are not sure if a list item applies to your research, read the appropriate section before selecting a response.

Materials & experimental systems

n/a	Involved in the study
☒	☐ Antibodies
☒	☐ Eukaryotic cell lines
☒	☐ Palaeontology and archaeology
☒	☐ Animals and other organisms
☒	☐ Clinical data
☒	☐ Dual use research of concern

Methods

n/a	Involved in the study
☒	☐ ChIP-seq
☒	☐ Flow cytometry
☒	☐ MRI-based neuroimaging
